# Wet Cough and Nasal Symptoms in Children: Can We Do Better?

**DOI:** 10.3389/fped.2019.00459

**Published:** 2019-11-26

**Authors:** Fernando M. de Benedictis, Ines Carloni, Pasquale Comberiati, Michael D. Shields, Andrew Bush, Anne B. Chang

**Affiliations:** ^1^Salesi Children's Hospital Foundation, Ancona, Italy; ^2^Department of Child and Mother Health, Salesi Children's Hospital, Ancona, Italy; ^3^Section of Paediatrics, Department of Clinical and Experimental Medicine, University of Pisa, Pisa, Italy; ^4^Centre for Experimental Medicine, Royal Belfast Hospital for Sick Children, Queen's University of Belfast, Belfast, United Kingdom; ^5^Department of Paediatric Respiratory Medicine, Imperial School of Medicine, National Heart and Lung Institute, Royal Brompton Hospital, London, United Kingdom; ^6^Child Health Division, Menzies School of Health Research, Casuarina, NT, Australia; ^7^Department of Respiratory and Sleep Medicine, Queensland Children's Hospital, Brisbane, QLD, Australia; ^8^Queensland University of Technology, Brisbane, QLD, Australia

**Keywords:** cough, protracted bacterial bronchitis, upper airway cough syndrome, nasal saline solutions, antibiotics

## Abstract

The causes of chronic cough in children are mainly dependent on the setting and age of the child. Protracted bacterial bronchitis is a frequent cause of morbidity in childhood, and antibiotic treatment is beneficial. Prompt recognition and early treatment is important both to prevent inappropriate use of asthma medications and also progression to bronchiectasis, but the diagnosis should not be made uncritically, because chronic wet cough is not necessarily due to lower airway disease. Upper Airway Cough Syndrome (UACS) is considered by some to cause chronic cough in childhood. Underlying UACS are many common conditions, including allergic rhinitis, adenoiditis and rhinosinusitis. Diagnosis relies on a combination of clinical criteria that are relatively sensitive but non-specific. The role of nasal endoscopy in children with chronic cough and signs suggesting UACS is unclear. Nasal saline solution irrigation is commonly used in UACS, but most studies have methodological biases, and efficacy data are scanty. Randomized controlled trials are urgently required. However, if saline washes, rather than oral antibiotics, can effectively treat some children with wet cough associated with upper airway conditions, antibiotic resistance could potentially be reduced. There is a need to further study wet cough and not to assume it to be equivalent to lower airway infection in all children.

All that coughs is not bronchitis

## Introduction

Cough is common in childhood (1). Spontaneous resolution of cough after an acute respiratory illness (ARI) typically occurs in 2–3 weeks, but occasionally children may cough longer (2). The British Thoracic Society (BTS) defined as chronic a cough lasting longer than 8 weeks (3), rather than the 4 weeks recommended by the American College of Chest Physicians (ACCP) (4) and the Thoracic Society of Australia and New Zealand (TSANZ) (5). In all, conclusions are predominantly based on observational data (6). The decision to include an intermediate time zone, defined as “prolonged acute cough” in the BTS guideline, was to allow a period for cough resolution for the minority of normal children who are still coughing with a simple cold after 2–3 weeks. However, no time duration is evidence based, and importantly, the decision to move to investigation is also critically determined by the general health of the child, and specifics of the cough; for example, if the history was strongly suggestive of inhaled foreign body, immediate investigation irrespective of cough is mandated.

This article aims to suggest a practical approach in children with wet cough and concomitant nasal symptoms through a vignette describing “a child” typical of those referred to outpatient services of two tertiary level Italian hospitals (Ospedale Materno-Infantile Salesi, Ancona, and Ospedale Santa Chiara, Pisa) by primary care pediatricians. The diagnostic work-up and the treatment options in this clinical setting are critically discussed.

## Case Presentation

A preschool boy presented with a 3-weeks history of a troublesome cough that had developed after an acute respiratory infection (ARI). Cough was initially dry, but gradually became wet. A runny nose, nasal obstruction and snoring at night were other features. He had received a 10-days course of oral amoxicillin-clavulanate with minimal effect. The family history was unremarkable. The personal history revealed frequent episodes of ARI during winter since starting attending day care. There was no history of wheeze. Parents were concerned about the frequency of respiratory episodes and had wished for “some tests” over the previous year. Routine laboratory tests including full blood count, erythrocyte sedimentation rate and C-reactive protein, were normal. Pharyngeal swab was also normal. Skin prick testing revealed minor sensitization to house dust mites. Physical examination revealed only a moist cough. A “rattle” sound was audible over the chest. The ear/nose/throat (ENT) examination revealed yellowish mucus dripping down into the oropharynx, a cobblestone pharyngeal mucosa and swollen turbinates. Nasal endoscopy revealed mild adenoidal hypertrophy with no evidence of sinus involvement. A diagnosis of adenoiditis was made and a 7-days course of nasal douching with hypertonic solution was prescribed. At 2-weeks follow-up, his cough had completely disappeared and physical examination was unremarkable. He remained well with no antibiotic therapy until a new ARI recurred after 2 months.

## Discussion

### Protracted Bacterial Bronchitis

The initial definition of protracted bacterial bronchitis (PBB) comprised a history of chronic wet cough, importantly a positive bronchoalveolar lavage (BAL) culture for a respiratory pathogen, and response to a 2-weeks course of oral amoxicillin-clavulanate (7). Since undertaking bronchoscopy and BAL in every child with chronic moist cough is unfeasible, the original definition was considered a microbiological one *(PPB-micro)* and new definitions were introduced (8). *PBB-clinical* eliminates the need for BAL, and amazingly, even a positive culture of lower airway secretions, but overtly acknowledges that other causes of chronic wet cough need to be excluded; in practice, absence of specific cough pointers: chest pain, history suggestive of inhaled foreign body, dyspnea, exertional dyspnea, hemoptysis, failure to thrive, feeding difficulties including choking/vomiting, cardiac or neurodevelopmental abnormalities, recurrent sinopulmonary infections, immunodeficiency, epidemiological risk factors for exposure to tuberculosis, signs of respiratory distress, digital clubbing, chest wall deformity, auscultatory crackles, chest radiographic changes other than perihilar changes, and lung function abnormalities. *PBB-extended* is PBB-micro or PBB-clinical, but cough resolves only after 4 weeks of antibiotics; and *recurrent-PBB* is >3 episodes PBB/year.

To confirm the clinical diagnosis of PBB clinicians have to rely on the duration (more than a completely arbitrary 4 weeks in most guidelines) and quality (wet or productive) of cough, and excluding other causes of wet cough. The proposed *PBB-clinical* definition lacks specificity and inherently has limitations. First, many cases of PBB may be “prolonged acute cough” lasting 4–8 weeks, which most commonly follows a viral ARI and typically resolves without intervention (9). Second, agreement between clinicians and parents for cough quality (dry vs. wet) in children is likely age-dependent, being better in children <2 years (10). Different types of cough can also be concurrent on many occasions (11). In children with post-ARI cough who received the diagnosis of PBB, parents reported cough as wet in 34%, dry in 26%, and variable in 16% of cases, and quality of cough was unknown in the remaining (12). Interestingly, the position statement of the Thoracic Society of Australia and New Zealand on cough in children emphasizes that wet cough actually may intermittently sound dry with minimal secretions (5); nevertheless, variable cough is usually classified as “wet cough.” Third, the clinical scenario is not always straightforward. Some children have recurrent wet cough secondary to a series of viral colds, which may coalesce in the mind of parents, making the diagnosis more difficult (13). Fourth, local differences in referral patterns may result in selection bias for the etiology of cough (14). Finally, modalities for excluding other causes of chronic cough in children with suspected PBB are not clearly defined.

The *PBB-clinical* definition may lead to substantial over-diagnosis and increased prescription of antibiotics if not meticulously verified. The evidence-base for benefits of antibiotic therapy in children with PBB mainly rely on a single randomized controlled study (RCT) involving children with chronic cough in Australia (15). Substantial concerns regarding the study methodology have been raised; chronic cough was defined as 3 rather than 4 weeks duration (but most children were coughing for far longer), prior therapies were not described, and the number of patients was small (16). Half of the patients were still coughing and 25% who received placebo were cough-free after 2 weeks; however, most patients who did not have clinical resolution had comorbidities (15). This suggests that prescription of antibiotics may be unnecessary in some cases or longer antibiotics may be required, as eventually the cough resolved in all patients after a further 2-weeks antibiotic treatment (ABC, personal report)—but *post-hoc* is not the same as *propter-hoc*.

Although two other RCTs were consistent that antibiotics are efficacious for resolving chronic wet cough (or at least, that cough resolved with the passage of time) (17), the optimal duration of treatment of PBB is unknown. In a retrospective chart review of 81 children diagnosed with PBB, 51% of patients were completely symptom-free after two courses of antibiotics, but 13% required six or more courses of antibiotics, or had continuous antibiotic prophylaxis for at least one winter to control symptoms (18). The ACCP guidelines (4) suggest 2 weeks (followed by 2 weeks if cough does not resolve), the BTS guidelines (3) indicate an initial course of 4–6 weeks, while the recent ERS Task Force (19) allows up to 4 weeks of treatment. For children with chronic wet cough without any specific pointer, the Chest Expert Cough Panel recommends that RCTs on the efficacy of different duration of antibiotics be undertaken in various clinical settings, particularly in primary care (20). What unfortunately appears to be misunderstood in primary care is that PBB means *chronic* cough. Yet in some settings, antibiotics are given for acute for which there is no indication.

### Upper Airway Cough Syndrome

Post-nasal drip (PND) is the drainage of secretions from the nose or paranasal sinuses into the pharynx. The suspicion of PND rests on the patient recognizing that something is dripping down into the throat and/or is suffering from frequent throat clearing. The finding of mucoid secretions in the pharynx or a cobblestone aspect of pharyngeal mucosa is suggestive.

The causal role of PND in chronic cough is a controversial issue both in adults (21) and in children (22), and the pathophysiologic mechanism is still debated (23). For these reasons, the ACCP committee has decided the term Upper Airway Cough Syndrome (UACS) should be used in preference to PND when discussing cough associated with upper airway conditions (24). UACS encompasses several conditions including post-ARI, rhinitis, rhinosinusitis (RS) and adenoiditis, with chronic cough being one of the main symptoms (25). Historically, the diagnosis of UACS has been determined by considering a combination of criteria including symptoms, physical examination and response to therapy with antihistamines. However, all of them are relatively sensitive but non-specific (26). The main clinical characteristics that may help distinguish PBB from rhinosinusitis and adenoiditis are shown in the Table 1.

**Table 1:** Main clinical characteristics of Protracted Bacterial Bronchitis and Upper Airway Cough Syndrome.

	**Protracted bacterial bronchitis (8)**	**Rhinosinusitis and adenoiditis (27)**
Cough	Chronic wet cough	Recurrent wet/dry cough
Age	Preschool children	Toddlers, preschool children
Personal history	Unremarkable	Day care attendance, recurrent URI
Season	All	Cold season
General appearance	Coughing	Running nose, nasal crease, allergic shiners
Snoring	Rare	Frequent
Voice	Normal	Nasal
Respiratory sound	Rattle over the chest	Coarse sound from the upper airway
ENT evaluation	Normal	Post-nasal drip, cobblestone pharyngeal mucosa
Allergy	Occasional	Frequent
Therapy	Response to antibiotics	Response to nasal cleaning

The reported incidence of UACS in children with chronic cough varies widely. Asilsoy et al. (28) used the ACCP criteria (4) to evaluate school-age children with chronic cough. Asthma (25%), PBB (23.4%) and UACS (20.3%) were the most common causes of cough. Using a different diagnostic algorithm including BAL, Marchant et al. (7) found UACS to be responsible for chronic cough only in a minority (3%) of preschool children when compared to PBB (40%). In a US study in school-age children who received a comprehensive workup including allergy skin tests, UACS (27.5%) and asthma (27.5%) were the most frequent causes of chronic cough, but validated tools for cough were not used (29). A Turkish study that used the BTS criteria (3) found UACS in 38% of school-age children, at least twice the frequency of PBB (18%) (30). A critical reappraisal of these studies suggests that the incidence of UACS in children is age-dependent and may be related to an ability of young children to describe symptoms. It should also be noted that a multicentre study in children with chronic cough showed that the younger the age group the more likely that PBB was the diagnosis (14).

The ACCP guidelines for adults explicitly state that the treatment options for UACS-induced cough are somewhat dependent on the subcategory of disease that is present (21). Antihistamines and nasal steroids have been recommended as ancillary treatment in patients with chronic RS (31), but the effect of these medications on cough is largely anecdotal (32). It is almost universal experience that viral colds are a cause of transient cough, which may be prolonged to an extent that is often not appreciated (33).

### Nasal Saline Solution Irrigation

Nasal saline solution irrigation (NSSI) has recently gained popularity in selected settings (34). An Italian survey among primary care pediatricians showed that most of them prescribe NSSI for both the prophylaxis and treatment of upper respiratory tract problems (35). However, NSSI does not seem to reduce the prescription of antibiotics, which is very high in Italian children (36). Isotonic saline (0.9%) and hypertonic saline (1.5% to 3%) solutions are commercial preparations commonly used (37). Disposable syringes with a proper nasal occlusion device or nasal douches (i.e., 20–30 ml each nostril) are the recommended techniques for administration (38).

The exact mechanism of action of NSSI is not known (or indeed, whether any effects are purely placebo), but mechanical cleaning of the nasal mucosa, which leads to the removal of inflammatory mediators and temporary improvement of mucociliary clearance, may be important. This may favor to resolve inflammation/infection spontaneously or with drugs.

Despite being used in daily practice, NSSI is only briefly mentioned in the guidelines for the treatment of RS (27, 39). Systematic reviews showed that NSSI was minimally effective in both acute upper respiratory tract infections (40) and chronic RS (41), but most studies have been conducted in adults or have important methodological problems (42). In a recent systematic review that evaluated the effect of NSSI in pediatric RS, only 1 out of 272 articles met all inclusion criteria (43). Demonstration of the efficacy of NSSI on reducing cough in patients with upper airway involvement is limited to pilot studies (44, 45).

## Conclusions

All children cough, but most of them are “expected” (i.e., post-viral) or merely a normal part of common childhood respiratory infections. Isolated dry cough in a community setting in an otherwise well child is unlikely to betoken any serious underlying condition. On the other hand, children with chronic wet cough often pose a diagnostic and management dilemma to the physician. Children similar to the one described in the vignette are frequently seen in primary care.

The clinical presentation of the case described does not fulfill the diagnostic criteria for PBB since the cough lasted only 3 weeks. Therefore, the prescription of antibiotics was inappropriate. Parents are often disturbed by cough and ask that “something is done.” In a prospective study in Australian children, a high proportion of parents thought antibiotics should be given for post-infectious cough (34%) or were unsure (24%); surprisingly, antibiotics were prescribed in 14% of episodes characterized by isolated dry cough with no fever (46). Post-infectious cough is difficult to distinguish from PBB and this may complicate therapeutic decisions (9). In a recent study on 839 children with cough following ARI, about 20% of patients still had cough on day 28 (12). A specific respiratory disorder was diagnosed in 84 of 117 children further evaluated by a pediatric pulmonologist, but the remaining 33 patients had non-specific cough. The case depicted in the vignette reinforces the message that extensive evaluation or treatment in most children with recurrent cough caused by ARI is usually unnecessary. On the contrary, the duration of antibiotic treatment is often too short in children who present clear symptoms of PBB.

The patient presented with a clinical history and physical signs consistent with UACS. There is no mention of nasal symptoms and/or signs in the recently extended list of cough pointers for the management of children with chronic wet cough (20). Some speculations need to be addressed. A low rate of UACS was reported only when ENT evaluation and allergy skin tests were not performed in the diagnostic workup (7, 47). Both investigations were included in studies reporting a high frequency of UACS (28, 29), confirming at least an association between allergy and upper airway involvement (48). In the Pediatric Allergy in America survey, a prevalence of 43% of sinus problems was found in children with AR compared with those without AR (4%) (49). Future research should be addressed to evaluate the role of allergy investigation in children with chronic cough.

In the vignette, the diagnosis of adenoiditis was made by nasal endoscopy. The inflammatory involvement of adenoids is common in children, especially those attending day care (50), as upper respiratory tract infections can easily spread to pharyngeal lymphoid tissue (51). Nasal endoscopy is a valuable tool for diagnosing adenoiditis and/or RS (27), and is well-accepted by children (52). However, the diagnostic role of this technique in children with suspected UACS needs to be evaluated in properly designed studies.

Cough in the above child had not disappeared after antibiotic therapy but did a few days after treatment with NSSI; however, spontaneous resolution cannot be excluded. In a prospective study, most children with suspected UACS had cough which resolved after treatment with NSSI (28). Cough is subject to the period- (spontaneous resolution) (11) and placebo-effect (53). The position statement on cough in children by the TSANZ (5) is appropriately cautious that any “positive” response in medication trials should not be assumed to be due to the medication. Although non-placebo intervention studies should be interpreted with caution, the clinical improvement in our case was obtained within the time-frame (2–3 weeks) that probably allows excluding the period-effect (6). Properly designed RCTs are however needed to confirm the efficacy of NSSI in such clinical setting and prove if antibiotic therapy may be unnecessary in some patients.

What is the role of the physician in this clinical setting? As a rule of thumb, we ought to spend much more time performing a careful history and diagnostic workup, and excluding serious conditions *before* prescribing an antibiotic; and this includes making the diagnosis of “normal child” and reassuring the family. Unfortunately, available data demonstrate that is difficult not to give in to this temptation. Respiratory diseases are the most common reason for the prescription of antibiotics in primary care, but many of them are unnecessary (54). Lack of alternative therapeutic interventions, and above all, the unwillingness to give an authoritative diagnosis of normal child, may increase demands by parents for antibiotics (55). Indeed, reduction of inappropriate treatment with antibiotics for prolonged cough is important to prevent patient harm and the risk of increased incidence of antimicrobial resistance in the community (56).

Some general advice may be useful. If the cough is dry and there are no specific alerts for a serious disease, a period of observation is all that is recommended. If the child develops a prolonged wet cough after a cold and no cough pointers are present, PBB should be considered. However, if nasal symptoms are present and the ENT investigation allows the exclusion of RS or rather confirms adenoiditis, a short trial with NSSI may be a reasonable option before considering antibiotic therapy. Indeed, although children with persistent wet cough have high rates of lower airway infection with bacteria and viruses, as documented by BAL findings, many of them have negative BAL (57).

The history of the management of children with persistent cough is fraught with wrong diagnoses, inappropriate treatments and uncritical clinical behaviors. There is no doubt that PBB is a real and significant cause of morbidity in childhood, and that prompt recognition and early treatment have represented a significant step forward to prevent the progression to bronchiectasis. However, we must learn a lesson from the past, when many children with chronic cough have been misdiagnosed and erroneously treated as asthmatics (58). Any uncritical enthusiasm in diagnosing PBB should be balanced with the risk of inappropriate therapy and the potential impact of antibiotics in selecting out resistant organisms. To avoid over diagnosis and unnecessary antibiotic use, we have to move rapidly beyond the umbrella term of PBB (59). Future research should incorporate additional diagnostic criteria (e.g., serum biomarkers as has been shown in community-acquired pneumonia?) (60) to better target therapy, and use robust endpoints to provide the definitive evidence of the true role of antibiotics in patients with chronic wet cough. RCTs are also urgent to determine the effectiveness of nasal douching in children with persistent cough and nasal symptoms. However, above all, clinicians need to be ready to make the diagnosis of “normal child” and reassure parents that treatment is not needed.

## Data Availability Statement

The datasets generated for this study are available on request to the corresponding author.

## Author Contributions

FB: original concept, drafting the manuscript, and final revision. PC and IC: help drafting the manuscript and references. MS, AB, and AC: critical reviewing of the manuscript. All authors approved the final version of the article.

### AMUPCG (Abruzzo Marche Umbria Pediatric Collaborative Group)

Ambrogi Maddalena (Gubbio), Amoroso Angelo (Pescara), Andreozzi Americo (Macerata), Basile Lucio (Pescara), Beghella Massimo (Ancona), Bellucci Nazareno (Todi), Bernacchi Stefania (Perugia), Bosco Fiorella (Pescara), Brachelente Linda (Perugia), Cappellucci Domenico (Pescara), Carnevale Maurizio (Pescara), Castiglione Giovanni Battista (Pescara), Chioccoloni Roberta (Marsciano), Coronati Valeria (Ancona), Della Vedova Giacomo (Spello), Di Filippo Antonella (Giulianova), Di Florio Rossana (Pescara), Di Giampietro Tiziana (Pescara), Di Girolamo Franca (San Benedetto del Tronto), Gabbanelli Gianna (Ancona), Gabriele Piera (Pescara), Gobbi Costantino (Macerata), Gonnellini Rita (Deruta), Guerrieri Arcangela (Ancona), Laguardia Luigi (Montesilvano), Liberati Marina (Ascoli Piceno), Martello Cecilia (Perugia), Merluzzi Angela (Città di Castello), Migliori Cesare (Ancona), Minucci Maria Giovanna (Foligno), Mora Marina (Ancona), Nuzzaci Roberto (Castelfidardo), Passali Maria Grazia (Pescara), Piermattei Loredana (Tolentino), Rossi Laura (Foligno), Ruggeri Anna Grazia (Matelica), Sardo Infirri Margherita (Spoleto), Solinas Lucia (Foligno), Truffarelli Francesca (Foligno).

### Conflict of Interest

The authors declare that the research was conducted in the absence of any commercial or financial relationships that could be construed as a potential conflict of interest.
